# Implementing paper-based patient-reported outcome collection within outpatient integrative health and medicine

**DOI:** 10.1371/journal.pone.0303985

**Published:** 2024-05-29

**Authors:** Roshini Srinivasan, Samuel N. Rodgers-Melnick, Rachael L. Rivard, Christine Kaiser, David Vincent, Francoise Adan, Jeffery A. Dusek

**Affiliations:** 1 Connor Whole Health, University Hospitals of Cleveland, Cleveland, OH, United States of America; 2 Duke University School of Medicine, Durham, NC, United States of America; 3 Department of Population and Quantitative Health Sciences, Case Western Reserve University School of Medicine, Cleveland, OH, United States of America; 4 Center for Evaluation Survey and Research, HealthPartners Institute, Minneapolis, MN, United States of America; 5 Department of Psychiatry, Case Western Reserve University School of Medicine, Cleveland, OH, United States of America; 6 Susan Samueli Integrative Health Institute, University of California Irvine, Irvine, CA, United States of America; 7 Department of Medicine, University of California Irvine, Irvine, CA, United States of America; Tehran University of Medical Sciences, ISLAMIC REPUBLIC OF IRAN

## Abstract

**Objective:**

To investigate the feasibility of pre- and post-encounter patient-reported outcome (PRO) measure collection within an outpatient integrative health and medicine (IHM) clinic and to characterize factors associated with successful completion.

**Methods:**

We conducted a retrospective review of 27,464 outpatient IHM encounters including 9,520 chiropractic; 8,237 acupuncture; 5,847 massage; 2,345 IHM consultation; and 1,515 osteopathic manipulation treatment encounters at four clinics offering IHM over 18 months. Patients were asked to complete paper questionnaires rating pain, anxiety, and stress from 0–10 immediately pre- and post-encounter. Generalized linear mixed effect regression models were used to examine the relationship between demographic, clinical, and operational covariates and completing (1) pre-encounter and (2) paired (i.e., pre and post) PROs.

**Results:**

Patients (*N* = 5587, mean age 49 years, 74% white, 77% female) generally presented for musculoskeletal conditions (81.7%), with a chief complaint of pain (55.1%). 21,852 (79.6%) encounters were among patients who completed pre-encounter PROs; 11,709/21,852 (53.6%) completed subsequent post-encounter PROs. Odds of PRO completion were more impacted by provider, operational, and clinical-level factors than patient factors. Covariates associated with increased odds of pre-encounter PRO completion included being female, having additional IHM encounters, and having a pain or anxiety complaint. Covariates associated with increased odds of paired PRO completion included being aged 31–40 vs. 51–60 years and having additional IHM encounters.

**Conclusion:**

Implementing a paper-based PRO collection system in outpatient IHM is feasible; however, collecting post-encounter PROs was challenging. Future endeavors should leverage the electronic health record and patient portals to optimize PRO collection and engage patients and clinical providers.

## 1.0 Introduction

Integrative health and medicine (IHM) is a whole-person paradigm of healthcare utilizing traditional, non-pharmacologic approaches to address the numerous physical and emotional factors which may impact an individual’s health [1]. Such an approach often combines therapeutic modalities from various ancient healing traditions [2], such as acupuncture, yoga, and massage, with conventional medical approaches, including pharmacotherapy and surgery, to provide care that addresses the mind, body, and spirit.

Applications of IHM in the ambulatory setting have been well-studied in the medical literature, from uses in the treatment of chronic pain [3] and symptom palliation [4, 5] to stress and anxiety reduction [6]. For example, a 2018 meta-analysis of 39 trials involving 20,827 patients found that acupuncture was superior to sham and/or no acupuncture treatment controls (p < 0.001) in the treatment of nonspecific musculoskeletal pain, osteoarthritis, chronic headache, or shoulder pain, with only a 15% decrease in effect at one year after treatment [7]. Similarly, results of a 2019 systematic review and meta-analysis of 47 randomized controlled trials (RCTs) suggest that chiropractic spinal manipulation therapy yields similar pain reduction as other recommended therapies (mean difference -3.17, 95% confidence interval [CI] -7.85 to 1.51) [8], and a lesser risk of benzodiazepine (OR 0.67; 95% CI 0.62 to 0.74) [9] or gabapentin (OR 0.53; 95% CI 0.40 to 0.71) prescription [10]. With regards to anxiety reduction, numerous systematic reviews support the effectiveness of acupuncture [11], massage [12], and yoga [13], as well as the safety and acceptability of these modalities.

Perhaps most notably, IHM has been widely utilized in the service of improving quality of life across populations and disease states [14]. A multi-site, prospective observational study of 409 patients undergoing an IHM program for chronic pain demonstrated significant improvements in pain (mean change -1.20; 95% CI − 1.60 to − 0.80) and quality of life (mean change -1.29; 95% CI − 1.54 to − 1.04) [15]. In a single-site prospective observational study, Crocker et al. reported a significant improvement (p = 0.002) in health-related quality of life from baseline to 12 months among 177 adults seeking ambulatory IHM care [16], as measured by numerous patient-reported outcome (PRO) instruments. PROs are subjective measures that quantify perceptions of health-related quality of life from the patient’s perspective [17]. Notably, these self-reported data are obtained directly from patients based upon their own appraisal of treatment impact [18], unlike physiological or laboratory assessments collected by clinicians or investigators. PROs are particularly vital in evaluating the effectiveness of health interventions for chronic conditions, where primary treatment goals may include improving quality of life, day-to-day function, and symptomatic treatment as well as direct disease management/reversal [19]. By providing data from the patient perspective, PROs capture holistic assessments of treatment effect that may otherwise not be obtained through traditional data collection procedures [20]. From a study design standpoint, routine collection of PROs in the clinical setting can highlight areas in need of methodological improvement [21].

Existing literature indicates that routine PRO collection and monitoring potentiates enhanced patient-clinician communication, clinician understanding of symptoms, symptom management, and quality of life [22]; thus, these inherently patient-centered measures complement conventional, objective outcomes, with comparable reliability between PROs and clinical measurements [23]. Routine implementation throughout the treatment course can help clinicians tailor and personalize care to each individual’s unique needs [24], while offering avenues for shared decision making, evaluating novel therapies and provider/treatment performance, and insight into systems-level quality improvement [25]. PRO collection has drastically increased over the last decade, with numerous professional societies encouraging their use in clinical care and research [26], especially in the midst of a shift towards value-based care reimbursement [25]. Within the realm of IHM, such measures may even constitute primary outcomes and the main indicators of treatment success, given IHM’s wholistic paradigm and focus upon quality of life [27, 28]: Thus, efforts to incorporate PRO collection into routine IHM care are vital in appropriately measuring care outcomes.

The utility of and implementation strategies for PRO collection have been widely studied in the outpatient setting, particularly in oncology and pain care [29], with a recent shift towards electronic data capture [30–32]. While electronic and paper-based methods have been shown to be comparable [33], electronic approaches pose certain limitations ranging from the individual to systems level. These limitations include the need for technology skills among patients and providers, internet access, significant financial investment, robust electronic health record (EHR) integration, and dedicated information technology support [30, 34]. In addition, challenges in technical accessibility may further widen the “digital divide,” contributing to existing disparities in PRO collection among diverse and underrepresented populations [35]. In contrast, though they require additional time and resources to print and transfer to electronic databases, paper-based PRO collection methods offer a uniquely low-tech, low-investment, accessible option.

Facilitators of successful outpatient PRO implementation across diverse fields of medicine have included: selecting clinically relevant PROs, integrating procedures within routine workflow, and minimizing complexity, while time constraints, poor communication around importance of PROs, language and/or technological literacy, and lack of administrative support have been barriers to completion [36, 37]. In an outpatient upper hand and extremity clinic, patient-level factors associated with completion of PRO measures included patient literacy, physical ability, and questionnaire design, among others [38, 39]. Prior research upon large-scale, longitudinal PRO collection within both the outpatient and inpatient IHM settings underscores its feasibility, acceptability, and potential benefit following routine implementation [16, 39–43]. A 2023 process-improvement study by Rodgers-Melnick et al. [44] highlighted efforts to optimize PRO collection by inpatient music therapists, including offering resources and guidelines for data collection as well as opportunities for staff feedback. However, to our knowledge, there is limited research on patient, clinical-, and operational-level factors associated with PRO collection across IHM modalities in the outpatient setting.

Successful integration of PRO measurement models into the outpatient IHM setting represents a critically important endeavor in measuring the real-world clinical effectiveness of IHM delivery. Thus, the present study reports on the pilot implementation of a paper-based PRO collection endeavor across four IHM clinics. The objectives of this study were to: (1) describe the implementation of a paper-based PRO collection system and (2) examine demographic, clinical, and operational characteristics associated with completing any pre-encounter PRO and completing a paired PRO (i.e., a post-encounter PRO following the completion of a pre-encounter PRO) among adult patients receiving outpatient IHM care.

## 2.0 Materials and methods

### 2.1 Participants and design

The present study is a retrospective review of all encounters among adult patients (ages 18 and older) receiving care at one of four outpatient IHM clinics between January 1, 2019 and July 31, 2020 which met the following criteria: (1) the encounter was for acupuncture, chiropractic, massage, integrative medicine consultation (IMC), or osteopathic manipulation treatment (OMT) and (2) the encounter was at an IHM clinic where patient service representatives (PSRs) had been instructed to administer the paper PRO measures. IHM encounters not meeting these criteria were excluded from the sample. Data access via the EHR began on March 16, 2020; raw data were extracted on May 17, 2023. Authors were able to access individually identifiable health data at time of data extraction, as approved by the hospital’s ethics committee.

### 2.2 Setting

University Hospitals (UH) is a not-for-profit health system in Northeast Ohio serving the needs of more than 1.2 million unique patients annually. University Hospitals Connor Whole Health (UHCWH) is a center offering IHM embedded within the larger UH health system. In accordance with prior established tenets of whole health, UHCWH partners with UH providers, departments, and institutes to provide IHM modalities and empower patients in managing health and wellbeing while centering patient goals [45]. In efforts to meet the growing demand for comprehensive strategies to manage a wide variety of health concerns, UHCWH services are integrated into the UH health system across primary care, multispecialty outpatient care [46], hematology/oncology [47–49], fertility, rehabilitation, and orthopedic divisions spanning a central academic medical center [50] and eight community hospitals [51].

IHM providers active during the study period included six acupuncturists, ten massage therapists, four chiropractors, four integrative medicine physicians and physician assistants, and one physician providing OMT across four clinical locations. Additionally, UHCWH had eleven expressive therapists (board-certified music and art therapists) and four mind-body program instructors who facilitated yoga therapy, mindfulness, meditation, and stress management and resilience training offerings.

### 2.3 Ethics and permissions

This study was approved by the UH Cleveland Medical Center Institutional Review Board (STUDY20200308) as a retrospective chart review with a waiver of informed consent by the hospital system’s research ethics committee. This study was conducted in accordance with the Declaration of Helsinki.

### 2.4 Data collection

#### 2.4.1 Questionnaires

The numeric rating scale (NRS) is commonly used for measuring pain, stress, and anxiety in clinical practice [52–54] and research [44, 48, 51], with demonstrated validity across these constructs. Owing to its simplicity, ease of use across populations, focus on symptoms in the present moment, and generalizability across various age and education levels [52], the NRS was selected as the instrument of choice in the present study.

A 3-item pre-encounter questionnaire was developed to measure pain, anxiety, and stress with space to rate each symptom on a numeric rating scale (NRS) from 0 (“none”) to 10 (“worst possible”), printed on a yellow-colored sheet of paper. An identical post-encounter questionnaire was also developed and printed on a blue-colored paper to be distinguished from the pre-encounter assessment. Brief acute NRS measures of pain, anxiety, and stress were specifically chosen given their successful collection in prior studies [51] and short completion time, minimizing response burden for patients. In addition, the selection of maximally relevant measures was prioritized in accordance with clinical practice guidelines [55], so as to integrate their collection within clinic workflow [38]. Furthermore, prior research has demonstrated that patients often seek IHM care for acute pain or psychosocial complaints, informing construct choice [56, 57]. As part of routine clinical care, the PSRs presented the pre-encounter questionnaires to patients as they checked in for their appointments, alongside any relevant intake information. Patients completed questionnaires by hand and subsequently presented these to the IHM provider. Following the IHM visit, providers presented patients with the post-encounter questionnaires and instructed patients to leave the completed questionnaire in the treatment room prior to leaving the clinic. Pre- and post-encounter NRS scores were then collected and entered into the EHR by the IHM clinical providers at the conclusion of the encounter. This workflow prevented patients from having to wait for PSRs to collect their completed questionnaires before leaving and promoted efficiency as the provider typically had the treatment note open to quickly complete data entry. Questionnaires were collected over an 18-month timespan.

The questionnaire collection processes were generally overseen by clinical operations directors (CK and DV), though there were no onsite PRO collection coordinators. Providers whose patients completed PRO measures in lower volumes were occasionally encouraged to increase collection by these coordinators. PRO collection efforts were de-prioritized, though not entirely stopped, at the onset of the coronavirus-2019 (COVID-19) pandemic in March 2020 and resumed in June 2020 so as to minimize the exchange of paperwork. During this time, acupuncture and massage operations paused entirely for six weeks, and chiropractic operated at a much lesser volume.

#### 2.4.2 Patient characteristics

The following data were extracted from all records which met eligibility criteria: (1) demographics including age, sex, race, and ethnicity; (2) International Classification of Diseases (ICD)-10 codes for all diagnoses and chief complaints listed in provider documentation; and (3) IHM documentation data of modality (i.e., acupuncture, chiropractic, massage, IMC, or OMT) and PROs (i.e., NRS scores of pain, anxiety, and stress) that were collected pre- and post-encounter. ICD-10 codes were only available for patients who had at least one encounter with an acupuncturist, chiropractor, physician, or advanced practice provider. Demographic data including sex, race, and ethnicity were extracted exactly as they were entered into the EHR by medical staff and may not have reflected the gender, racial, and/or ethnic identities of the patients included in this study [58]. All data, including full note documentation, were extracted via a single structured query language script from the UH Electronic Data Warehouse. Regular expressions functions including str_extract_all from the stringr package [59] and regmatches, gregepr, sub, and gsub from base R version 4.3.0 were used to extract clinical information from the free-text note.

### 2.5 Statistical analysis methods

Descriptive statistics were tabulated to characterize the study population, while run charts were constructed to visualize PRO questionnaire completion rates per week. Generalized linear mixed effect regression models with binary outcome distribution were used to examine the relationship between demographic, clinical, and operational characteristics with each of the two outcomes: (1) completion of any pre-encounter PRO, and (2) paired PRO completion which was defined as completing a post-encounter PRO following the completion of a respective pre-encounter PRO (e.g., pre-encounter stress and post-encounter stress). Both models included the following covariates: (1) demographics including age (18–30, 31–40, 41–50, 51–60, 61–70, or 71+), sex (male or female), ethnicity (non-Hispanic [NH], Hispanic/Latino, or declined/missing), and race (White, American Indian, Asian, Black/African American, other/multi-racial, or declined/missing; (2) clinical characteristics including chief complaints (yes or no) of pain, headache, or anxiety and number of visits during the study period; and (3) operational characteristics including clinic location and time period (2019 quarter [Q] 1 through 2023Q3). Both models included a random intercept for patient and a random residual effect for provider nested within modality. The second model was limited to only those who provided a pre-encounter score and included a covariate for whether any pre-encounter PRO was rated ≥4 (i.e., moderate-to-severe pain, stress, or anxiety). The general linear mixed model was generated using proc GLMIMMIX in SAS software, Version 9.4 of the SAS System for Windows (Cary, NC).

## 3.0 Results

### 3.1 Sample

Between January 2019 and July 2020, there were 27,464 IHM encounters among 5,587 patients receiving outpatient care at UHCWH. These 27,464 included encounters for chiropractic (34.7%), acupuncture (30.0%), massage (21.3%), IMC (8.5%), and OMT (5.5%).

### 3.2 Demographics, chief complaints, and utilization

**Table 1** summarizes demographics, chief complaints, and utilization characteristics. Patients (mean age: 48.95 ± 15.86 years at first encounter) were mostly White (74.0%) or Black/African American (17.0%), non-Hispanic (85.8%), and female (77.0%). Over half of patients reported a chief complaint of pain (55.1%) at some point during the study, with specific pain complaints located in the back (34.3%), neck (23.4%), and shoulder (23.4%). Other common chief complaints included stress (12.7%), anxiety (11.3%), and headache/migraine (8.9%). Median [IQR] encounter engagement volume among patients was 2 [1–6] encounters, with the most common IHM modality being massage (35.8%) followed by acupuncture (28.2%), chiropractic (27.5%), IMC (23.1%), and OMT (13.0%).

**Table 1:** Patient demographics, chief complaints, and utilization characteristics.

Variable	N = 5,587
**Patient age (years), mean ± SD**	48.95 ± 15.86
**Patient age (years), median [range]**	49.00 [18.00, 98.00]
**Sex, n (%)**	
Female	4,300 (77.0%)
Male	1,287 (23.0%)
**Race, n (%)** ^**a**^	
White	4,135 (74.0%)
Black/African American	950 (17.0%)
Declined, Missing, or Unknown	343 (6.1%)
Other Race/Multi-Racial	78 (1.4%)
Asian	71 (1.3%)
American Indian	10 (0.2%)
**Ethnicity, n (%)**	
Non-Hispanic	4,792 (85.8%)
Declined, Missing, or Unknown	656 (11.7%)
Hispanic or Latino	139 (2.5%)
**Pain complaint, n (%)**	3,077 (55.1%)
Back pain complaint, n (%)	1,917 (34.3%)
Neck pain complaint, n (%)	1,307 (23.4%)
Shoulder pain complaint, n (%)	1,307 (23.4%)
**Headache/migraine complaint, n (%)**	500 (8.9%)
**Tension complaint, n (%)**	935 (16.7%)
**Stress complaint, n (%)**	712 (12.7%)
**Anxiety complaint, n (%)**	632 (11.3%)
**Total treatments, median [IQR]**	2.00 [1.00, 6.00]
Received acupuncture, n (%)	1,573 (28.2%)
Received chiropractic, n (%)	1,535 (27.5%)
Received IMC, n (%)	1,291 (23.1%)
Received massage, n (%)	1,999 (35.8%)
Received OMT, n (%)	725 (13.0%)

^a^Race, including multi-racial, is reported exactly as it was entered into the EHR: Abbreviations: EHR, electronic health record; IMC, integrative medicine consult; IQR, interquartile range; OMT, osteopathic manipulative treatment; SD, standard deviation.

### 3.3 Clinical characteristics

**S1 Table** summarizes the Major Expanded Diagnosis Clusters (MEDCs) among 4,194 patients who attended at least one IHM encounter that was not massage. Most patients presented for musculoskeletal diagnoses (81.7%), followed by neurologic diagnoses (50.5%) such as headaches, sleep problems, and migraines. Many patients also presented for general signs and symptoms (49.5%), including fatigue (9.9%), administrative concerns (25.3%) such as non-specific lab abnormalities (22.1%) and preventive care (6.7%), and psychosocial and mental health (23.6%) conditions.

### 3.4 Pre-encounter PRO completion data

**Table 2** summarizes pre-encounter PRO completion characteristics among all encounters including (1) the count and percentage of encounters with complete pre-encounter PROs, (2) the count and percentage of encounters with pre-encounter PROs rated 0/10 (i.e., no symptom), and (3) the count, percentage, mean, and standard deviation (SD) of encounters with pre-encounter PROs rated ≥1/10 (i.e., mild to severe symptoms). Of the 27,464 encounters included in this sample, 21,852 (79.6%) were among patients who completed at least one PRO. Of the 21,852 encounters in which any pre-encounter PRO was completed, the majority were obtained from chiropractic encounters (41.2%), followed by acupuncture (25.8%), massage (20.4%), IMC (7.5%), and OMT (5.1%) encounters.

**Table 2:** Pre-encounter PRO collection characteristics.

Variable	Modality	Total N	Complete Pre-Encounter PRO N (%)	Pre-Encounter PRO = 0 N (%)	Pre-Encounter PRO ≥ 1 N (%)	Pre-Encounter PRO ≥ 1 Mean ± SD
Pre-encounter pain	All	27464	21798 (79.4%)	2046 (9.4%)	19752 (90.6%)	4.25 ± 2.25
	Chiropractic	9520	8983 (94.4%)	255 (2.8%)	8728 (97.2%)	4.5 ± 2.31
	Acupuncture	8237	5622 (68.3%)	847 (15.1%)	4775 (84.9%)	3.94 ± 2.16
	Massage	5847	4454 (76.2%)	434 (9.7%)	4020 (90.3%)	3.94 ± 2.08
	IMC	2345	1637 (69.8%)	391 (23.9%)	1246 (76.1%)	4.72 ± 2.42
	OMT	1515	1102 (72.7%)	119 (10.8%)	983 (89.2%)	4.2 ± 2.17
Pre-encounter stress	All	27464	19749 (71.9%)	3373 (17.1%)	16376 (82.9%)	4.02 ± 2.4
	Chiropractic	9520	7118 (74.8%)	1484 (20.8%)	5634 (79.2%)	3.99 ± 2.48
	Acupuncture	8237	5496 (66.7%)	844 (15.4%)	4652 (84.6%)	3.83 ± 2.31
	Massage	5847	4412 (75.5%)	650 (14.7%)	3762 (85.3%)	4.05 ± 2.35
	IMC	2345	1623 (69.2%)	183 (11.3%)	1440 (88.7%)	4.67 ± 2.5
	OMT	1515	1100 (72.6%)	212 (19.3%)	888 (80.7%)	3.92 ± 2.29
Pre-encounter anxiety	All	27464	19767 (72%)	4814 (24.4%)	14953 (75.6%)	3.76 ± 2.32
	Chiropractic	9520	7121 (74.8%)	1980 (27.8%)	5141 (72.2%)	3.81 ± 2.43
	Acupuncture	8237	5496 (66.7%)	1185 (21.6%)	4311 (78.4%)	3.65 ± 2.22
	Massage	5847	4429 (75.7%)	1068 (24.1%)	3361 (75.9%)	3.54 ± 2.2
	IMC	2345	1627 (69.4%)	261 (16%)	1366 (84%)	4.44 ± 2.43
	OMT	1515	1094 (72.2%)	320 (29.3%)	774 (70.7%)	3.7 ± 2.25

Mean ± SD are of patient-reported outcomes ≥ 1. Abbreviations: IMC, integrative medicine consult; SD, standard deviation

Of the 27,464 encounters, 21,798 (79.4%) were among patients who completed a pre-encounter pain questionnaire. Across 19,752 encounters in which patients reported pain ≥1, patients reported mean (± SD) pain scores of 4.25 ± 2.25 units on the NRS. Similarly, 19,749 (71.9%) encounters were among patients who completed a pre-encounter questionnaire for stress with mean ± SD scores of 4.02 ± 2.40 among patients reporting stress ≥1. Lastly, 19,767 (72%) encounters were among patients who completed a pre-encounter questionnaire for anxiety with mean ± SD scores of 3.76 ± 2.32 among patients reporting anxiety ≥1. **Table 2** provides additional details regarding pre-encounter pain, stress, and anxiety scores by modality.

**Fig 1:**
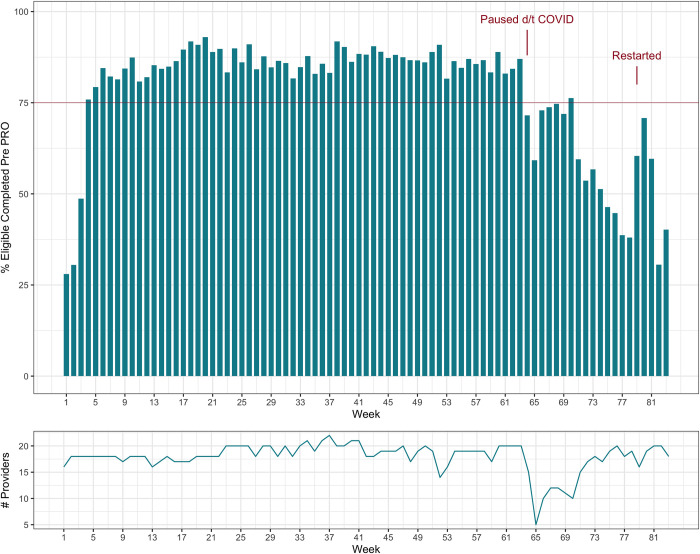
Run chart of pre-encounter PRO completion.

A run chart of pre-encounter PRO completion over the 18-month (83-week) period is shown in **Fig 1**. Notable features include a decrease in net pre-encounter PRO completion at week 64 due to de-prioritizing data collection in the wake of COVID-19-related infection control concerns, as well as an increase at week 79 when operational emphasis upon paper PRO collection resumed. The median [IQR] percentage of pre-encounter PRO completion per week was 84.6% [74.3–87.5] over 83 weeks. Pre-encounter PRO completion was maintained at above 75% of all encounters per week for 61/83 (73.5%) weeks.

**Fig 2:**
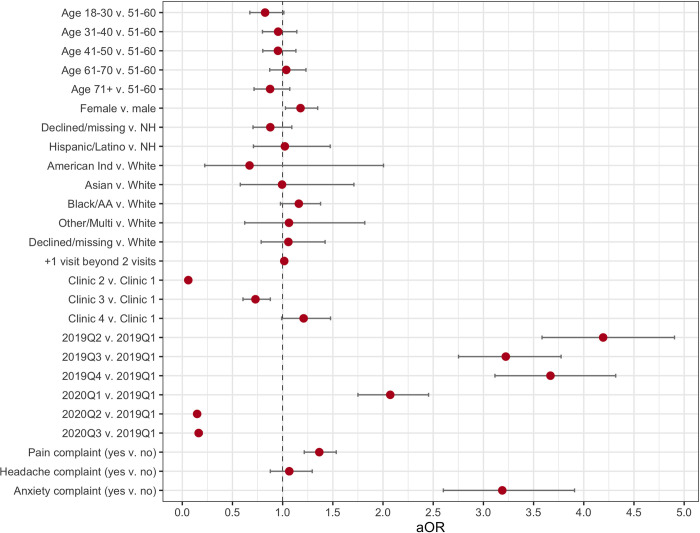
Adjusted odds ratios (aOR) for completing any pre-encounter PRO questionnaire.

**Fig 2** and **S2 Table** depict adjusted odds ratios (aOR) for completing any pre-encounter PRO questionnaire. Chief complaints of pain, headache, and anxiety were selected due to their high clinical relevance and prevalence so as to display in the model. For this generalized linear mixed model with binary distribution, the covariance parameter estimate ± standard error (SE) for the random effect of provider nested within modality was 3.25 ± 0.96, while the covariance parameter estimate for the random effect of patient was 0.96 ± 0.06. Among all 27,464 encounters, the following were associated with increased odds (aOR [95% CI]) of completing a pre-encounter PRO: (1) being female as compared to male (1.178 [1.028, 1.349]); (2) attending an additional IHM encounter beyond two encounters during the study period (1.016, [1.009, 1.022]); (3) being seen in 2019Q2 (4.193 [3.584, 4.904]), 2019Q3 (3.223 [2.752, 3.774]), 2019Q4 (3.668 [3.115, 4.318]) or 2020Q1 (2.072 [1.750, 2.454]) as compared to 2019Q1; and (4) having a pain (1.365 [1.215, 1.534]) or anxiety complaint (3.188 [2.601, 3.908]). Covariates associated with decreased odds of completing a pre-encounter PRO included (1) being seen in clinic 2 (0.060 [0.047, 0.075]) or clinic 3 (0.729 [0.605, 0.877]) as compared to clinic 1 and (2) being seen in 2020Q2 (0.148 [0.124, 0.177]) or 2020Q3 (0.163 [0.133, 0.199]) as compared to 2019Q1.

### 3.5 Paired PRO completion data

Of the 21,852 encounters in which patients completed at least one PRO, 11,709 (53.6%) were among patients who completed a subsequent post-encounter PRO.

**Fig 3:**
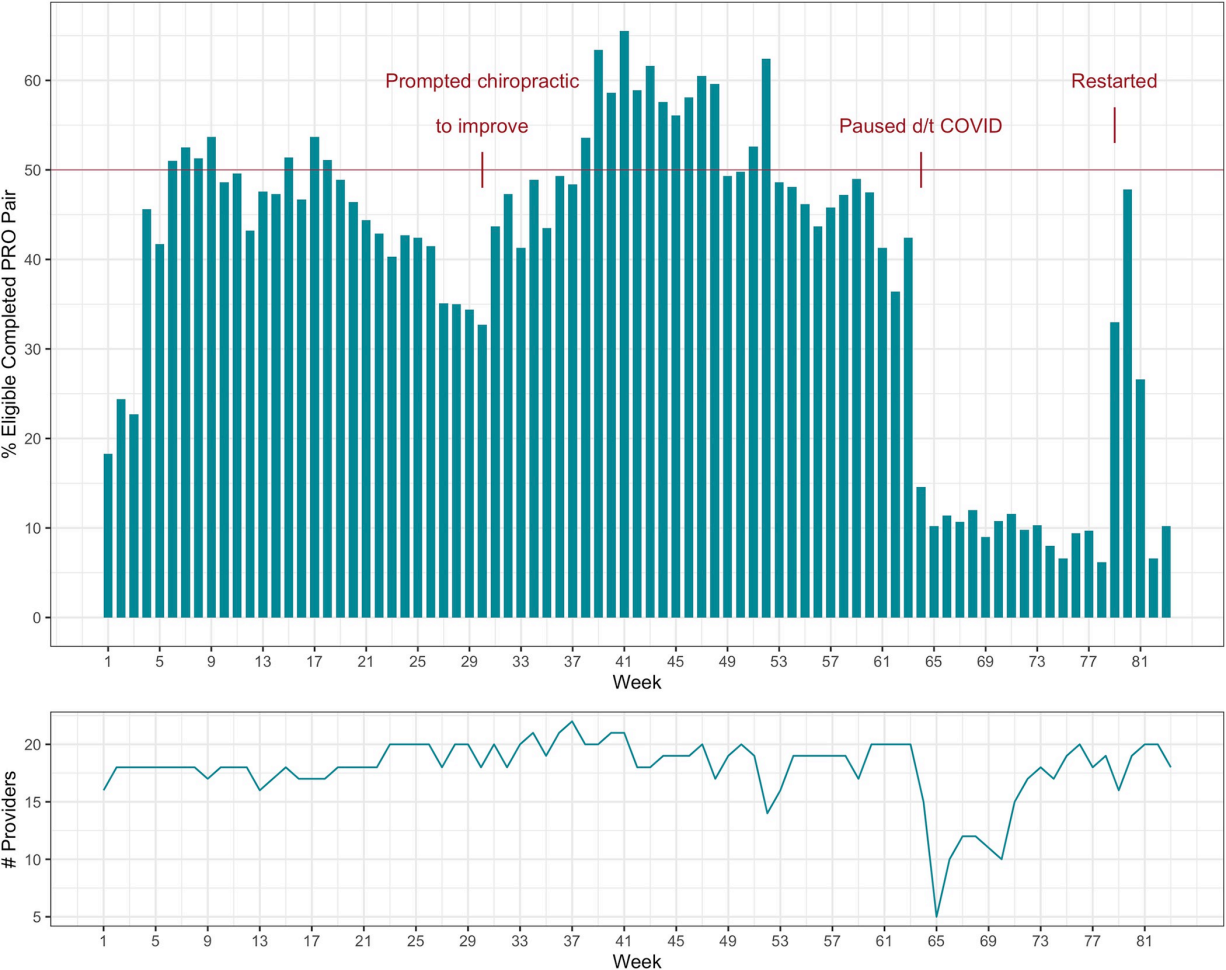
Run chart of paired PRO completion.

**Fig 3** depicts a run chart of paired PRO completion. Notable features include a sharp increase in paired PRO completion at week 30 when the chiropractic service began increasing efforts to obtain post-encounter PROs, a decrease at week 64 due to infection control concerns related to COVID-19, and an increase at week 79 when paper PRO collection efforts were resumed. The median [IQR] percentage of paired PRO completion per week was 45.6 [29.7–49.7]. Across 83 weeks, only 20 (24.1%) were weeks in which paired PRO completion constituted ≥50% of all encounters.

### 3.6 Model 2: Predicting post-encounter PRO completion

**Fig 4:**
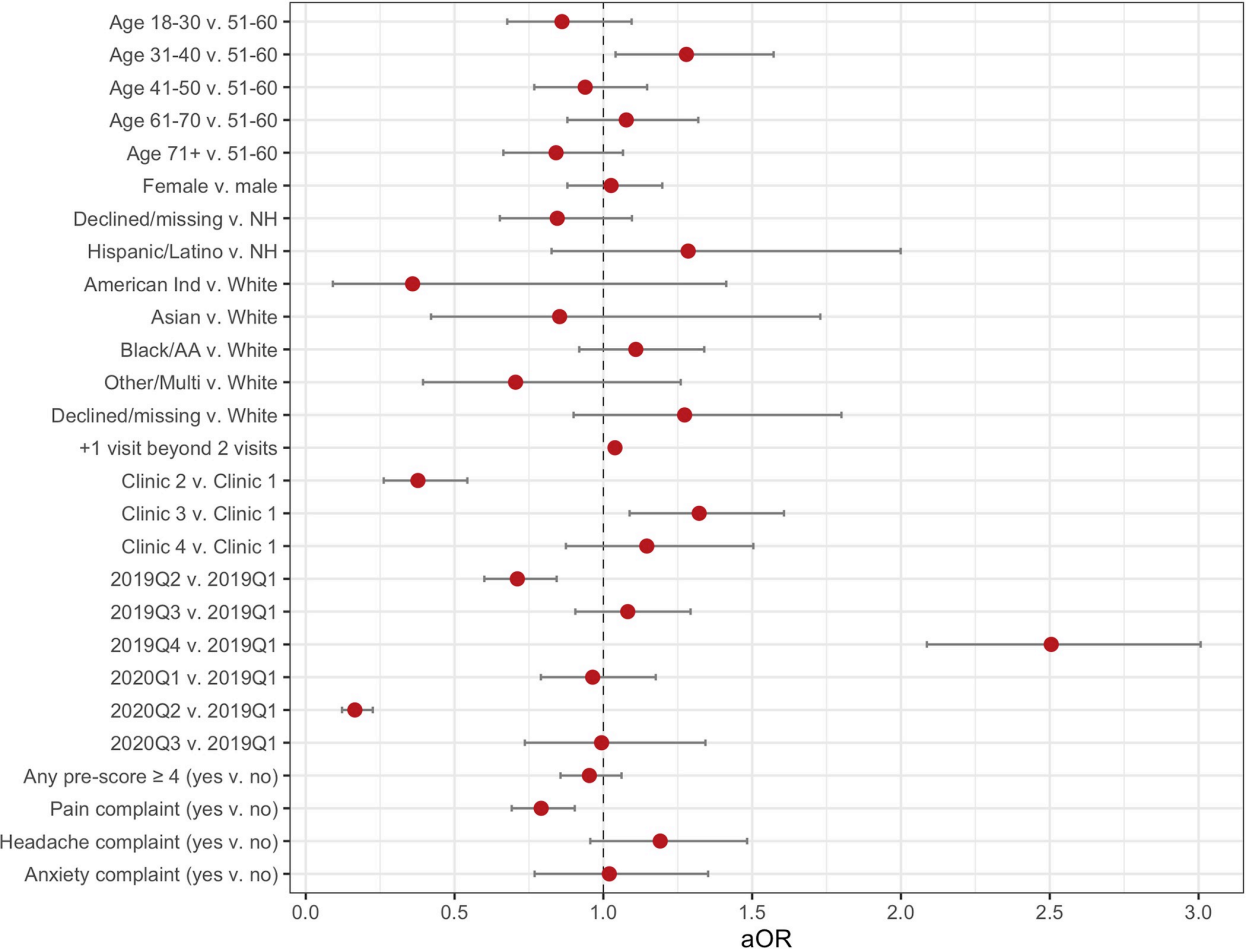
Adjusted odds ratios (aOR) for completing any post-encounter PRO questionnaire following the completion of a pre-encounter PRO questionnaire.

**Fig 4** and **S3 Table** depict adjusted odds ratios (aOR) for completing any post-encounter PRO questionnaire following the completion of a pre-encounter PRO questionnaire. For this model, the covariance parameter estimates for the random effect of provider nested within modality was 8.80 ± 2.77, while the covariance parameter estimate for the random effect of patient was 1.36 ± 0.08. Among 21,852 encounters, the following were associated with increased odds (aOR [95% CI]) of completing a post-encounter PRO: (1) being aged 31–40 years as compared to 51–60 years (1.279 [1.041, 1.572]); (2) having an additional IHM encounter beyond 2 encounters (1.039, [1.031, 1.047]); (3) visiting clinic 3 as compared to clinic 1 (1.322 [1.088, 1.607]); and (4) being seen in 2019Q4 as compared to 2019Q1 (2.505 [2.087, 3.007]). Covariates associated with decreased odds of completing a post-encounter PRO included (1) visiting clinic 2 as compared to clinic 1 (0.377 [0.262, 0.543]); (2) being seen in 2019Q2 as compared to 2019Q1 (0.711 [0.600, 0.843]); and (3) having a pain complaint (0.791 [0.692, 0.904]).

## 4.0 Discussion

The purpose of this study was to describe the implementation of a paper-based PRO collection system within an outpatient IHM center and determine characteristics associated with completion of pre-encounter and paired (e.g., pre- and post-encounter) measures. Overall, pre-encounter PRO collection was feasible and successful, while a smaller proportion of paired PROs were collected. Provider- and organization-level factors appeared to impact odds of PRO completion more than patient-level factors.

The demographic distribution of patients in this study is consistent with previous literature on IHM utilization [60], reflecting predominantly white, middle-aged females seeking IHM care; however, 17% of patients in this study identified as Black, representing a higher proportion as compared to other IHM literature [16, 61]. Black or African-American individuals represented 29% of the total population of the county in which the IHM clinics were located per the 2020 United States census [62]. Thus, this work continues to underscore the need to diversify IHM clientele through innovative outreach and addressing barriers, both individual and systemic, to equitable IHM access and participation.

The observed prevalence of pain complaints and musculoskeletal conditions is consistent with the literature that such complaints, including back and neck pain, are the most common reasons cited to seek IHM care [63, 64]. Thus, further investigation of PROs is warranted to demonstrate both immediate and longitudinal impacts of IHM for these conditions.

The most prevalent IHM modality sought among all patients was massage (35.8%), which may be due to initial massage therapy treatments being offered free-of-charge to employees within the UH health system during a period within the study timeframe. These rates are consistent with previous work demonstrating that massage is one of the most common IHM modalities sought across demographic groups [56]. Notably, at the time of study, massage was an exclusively self-pay modality, where others had some level of insurance reimbursement depending upon patient type and associated diagnoses. It may be worthwhile for future endeavors to examine the relationship between insurance status and modalities sought among patients seeking IHM care.

Among the key observations of this pilot study was the sheer volume able to be captured using a low-tech, paper-based PRO collection system. The large sample size of 27,464 encounters within this timeframe, from which 21,852 pre-encounter PRO questionnaires were completed, is akin to that of Hui et al. [30], who surveyed 6631 patients across 25,767 visits to an outpatient palliative care clinic. In their study, PROs were completed by 100% of patients, attributable to mandatory completion via integration of both paper and electronic versions into clinic workflow, follow-up by clinic nurses, and clinician utilization of PROs during the visit. In contrast, patients presenting to any of the four IHM clinics where PSRs were collecting these data were not required to complete PROs; our observed median 84.6% pre-encounter PRO collection exceeds the average 71% response rates of PROs captured by clinical registries [57]. Within the realm of outpatient PRO implementation, our pre-encounter collection rate falls within the range of other studies where it was not mandatory, ranging from 54% to 88% [65–67], and exceeds the 59% collection rate across 12,207 visits within an ambulatory IHM clinic [39]. However, to our knowledge, this study is one of the first to explore PRO collection immediately pre- and post-encounter within IHM modalities. From our perspective, the large volume of data capture, particularly pre-encounter, is attributable to support from operations staff and training of PSRs, the low-tech, ease of disseminating paper questionnaires, and responsiveness of clinical staff to feedback regarding increasing collection efforts.

Demographic and clinical covariates associated with increased odds of pre-encounter PRO collection included female sex, attending more than two IHM encounters, and having a pain or anxiety complaint. This is consistent with previous research in which females were more likely to engage with health information, such as contributing to survey responses [68, 69].

Additionally, patients presenting with pain or anxiety complaints may have been more likely to complete pre-encounter PRO measures out of a willingness to communicate the intensity of their needs to providers, as well as the relative heterogeneity of symptoms appraised as such [70].

Decreases in both pre-encounter and paired PRO collection at week 64 corresponded to operational pauses due to the onset of COVID-19: clinical operations were greatly reduced, and less emphasis was placed upon PRO collection, though it was not eliminated entirely. Anecdotally, PSRs noted that hesitance towards touching clinic writing instruments due to perceived risk of infection was a major deterrent to PRO questionnaire completion, though infection has been shown to be unlikely from day-to-day use of droplet-contaminated fomites [71]. However, these observations contextualize findings of decreased odds of pre-encounter completion during 2020Q2 and 2020Q3, when the COVID-19 pandemic first began to affect clinical operations within the health system.

In contrast, paired PRO completion rates (53.6% of those in which pre-encounter PROs were collected) were lower than pre-encounter PRO completion rates (79.6% of all encounters). To our knowledge, limited existing work describes immediate post-encounter PRO collection in the IHM setting; however, using Sisodia et al.’s [72] threshold of 50% completion as a successful collection rate, we achieved successful paired PRO collection during just 24.1% of the study period. This observation corroborates the need for future, targeted efforts to optimize immediate post-encounter PRO collection, particularly leveraging electronic data capture methods delivered via text message.

Covariates associated with increased paired PRO collection included being aged 31–40 years versus 51–60 years, attending more visits to the IHM center, and presenting to IHM care at Clinic 3. Though patients with a pain complaint were more likely to complete a pre-encounter, they were less likely to complete a paired PRO. Though the exact reasoning for this is unclear, possible explanation include pain taking more time to resolve than anxiety, it being a comparatively physical measure compared to anxiety or stress, or electing to verbally share pain relief with the provider rather than completing an NRS.

Clinic-to-clinic differences in rates of PRO collection are most likely attribute to operational differences in PSR staff. In particular, clinics 3 and 4 were locations shared by many departments in addition to the whole health center. Thus, PSRs at these locations, were managing patients from numerous providers, and may not have had direct, consistent communications from the whole health center’s policies or initiatives, such as reminders or emphasis upon questionnaire administration. In addition, these locations typically had low-patient-volume clinical operations. While real-time process monitoring was not conducted in this study, providing a comprehensive PRO collection training, managerial expectation, and monitoring process represents an important opportunity to unify administrative efforts to optimize and equalize collection across sites [38, 39, 44].

Notably, while massage was the most prevalent modality, followed by acupuncture, then chiropractic, pre-encounter PROs were collected most from chiropractic, then acupuncture, then massage therapy encounters. Differences in PRO collection by modality may be attributed to various factors. First, while we did not have a designated PRO collection coordinator, we did have administrative champions driving these efforts within the acupuncture (CK) and chiropractic (DV) modalities. Further, the responsivity of clinical staff to encouragement to increase PRO collection, evidenced in the increase in paired PRO completion at 30 weeks, underscores the importance of real-time monitoring and coordination to maximize data collection. In addition, other factors such as differences in workflows between providers and modalities may contribute to these observed differences in pre-encounter PRO collection. Thus, efforts to optimize PRO collection must account for modality-specific considerations.

The covariance parameter estimates from both logistic regression models suggest that provider- and operational-level factors, such as provider behavior, clinic location, and timing, were more influential than patient-level factors on PRO collection outcomes. This is consistent with prior findings that physician and administrative engagement were most strongly associated with PRO collection and program success [72]. Increased odds of completion of any PRO questionnaire at later quarters of 2019 may reflect the “adjustment time” to successfully integrate a PRO collection system into clinical operations [30]. Notably, neither race nor age had a detectable impact on odds of pre-encounter PRO completion.

Variation in PRO completion rates between clinic locations may be attributable to different PSR staffing each site, as well as each clinic’s unique operational procedures when patients checked in for their visit, underscoring the pivotal role of PSR and operations engagement in successful administration. In addition, this observation highlights the integral role of administrative and clinical staff engagement in PRO collection endeavors, and that clinic-specific factors must be considered with designing strategies for PRO implementation–i.e., there may not be a “one size fits all” approach [22].

This study had several limitations. These included potential response bias (i.e., non-assessment of other variables that may account for response rates) and lack of privacy for patients completing paper-based measures, which may have influenced patients who may have otherwise not completed a PRO to do so, or vice versa. In addition, the fact that we did not closely monitor PRO collection in real-time limited our ability to identify real-time barriers to PRO collection and generate solutions. Future implementation strategies should ideally include efforts to increase patient privacy while having dedicated personnel to manage and monitor real-time PRO collection efforts and aid patients in completing forms as needed. This latter point may be crucial as PRO collection migrates to an electronic format, especially to avoid worsening existing disparities.

In addition, the studied characteristics were just a snapshot of the myriad factors that may be associated with PRO completion, and it is near impossible to capture all attributes. However, this research provides an important initial framework for characterizing at least some factors associated with PRO completion in the IHM setting. While creating an abbreviated questionnaire measuring pain, anxiety, and stress allowed for an easy-to-implement instrument, our inability to measure other constructs (e.g., nausea or fatigue) for which patients also commonly seek IHM constitutes another study limitation.

Strengths of this study included its large sample size, implementation across four IHM clinic settings within a large healthcare system, and the novel collection effort of PROs immediately before and after IHM encounters. This represents a unique, large-scale effort to characterize PRO completion (and subsequently, perceived treatment impact) immediately following IHM modalities. The utilization of real-world data from the EHR allowed us to extract variables from many clinical encounters without having to enter this information into a separate database during the study. In addition, the relatively low-tech paper-based data collection method allowed for easy integration into clinic workflow, avoiding the logistical and access-related issues that electronic collection may pose.

With high rates of completion for pre- and paired measures, the low-cost paper questionnaires proved feasible (e.g., established proof of concept) from the lens of a single-encounter collection. However, electronic collection would be better suited for longitudinal collection and data analysis, especially if integrated into the EHR. Thus, future research will ideally explore routine electronic PRO collection and implementation in the outpatient IHM setting. Such efforts would align with current trends, especially in the post/chronic COVID-19 pandemic era, to move toward electronic data capture and potentiate real-time collection and visualization for both patients and providers [30]. If issues of implementation are surmounted, electronic PRO collection can improve data accuracy and integrity [73], maximizing compliance while minimizing secondary data entry errors [74] and administrative burden [75, 76]. In fact, the present work has inspired the subsequent piloting of efforts at the authors’ own institution to move toward similar methods of electronic data collection.

## 5.0 Conclusions

Implementation of a paper-based immediate PRO collection system in the outpatient IHM setting is feasible. Overall, collection of pre-encounter PROs was successful, while a smaller proportion of paired PROs were collected. Provider- and organization-level factors appeared to impact rates of any (pre- or paired) PRO completion more than patient-level factors.

## Supporting information

S1 Table Major Expanded Diagnosis Clusters (MEDCs) of patients attending at least one non-massage IHM encounter. (DOCX)

S2 Table Adjusted odds ratios (aOR) for completing any pre-encounter PRO questionnaire. (DOCX)

S3 Table Adjusted odds ratios (aOR) for completing any post-encounter PRO questionnaire following the completion of a pre-encounter PRO questionnaire. (DOCX)
