# Supplementary material for: Implementing paper-based patient-reported outcome collection within outpatient integrative health and medicine
Source: PLoS One. 2024 May 29;19(5):e0303985. doi: 10.1371/journal.pone.0303985 (PMC11135778; doi:10.1371/journal.pone.0303985)
Supplement: S1 Table — (DOCX) [file pone.0303985.s001.docx]

| **Supplemental Table 1: Expanded Diagnosis Clusters** | |
| --- | --- |
| **MEDC** | **N = 4,194** |
| **Musculoskeletal** | 3425 (81.7) |
| Musculoskeletal other | 2644 (63) |
| Low back pain | 1940 (46.3) |
| Musculoskeletal signs and symptoms | 1751 (41.8) |
| Cervical pain syndrome | 1241 (29.6) |
| **Neurologic** | 2117 (50.5) |
| Neurologic signs and symptoms | 1414 (33.7) |
| Headaches | 456 (10.9) |
| Sleep problems | 233 (5.6) |
| Migraines | 231 (5.5) |
| **General signs and symptoms** | 2078 (49.5) |
| Non-specific signs and symptoms | 1864 (44.4) |
| Debility or undue fatigue | 414 (9.9) |
| **Administrative** | 1060 (25.3) |
| Administrative concerns and non-specific lab abnormalities | 927 (22.1) |
| Preventive care | 279 (6.7) |
| **Psychosocial and mental health** | 990 (23.6) |
| Anxiety | 613 (14.6) |
| **Nutrition** | 600 (14.3) |
| **Cardiovascular** | 378 (9) |
| **Gastrointestinal or hepatic** | 306 (7.3) |
| **Female reproductive** | 303 (7.2) |
| **Endocrine** | 245 (5.8) |
| **General surgery** | 234 (5.6) |
| **Skin** | 210 (5) |
| **Respiratory** | 209 (5) |
| **Rheumatologic** | 120 (2.9) |
| **Ear, nose, and throat** | 108 (2.6) |
| **Allergy** | 83 (2) |
| **Malignancies** | 72 (1.7) |
| **Genito-urinary** | 66 (1.6) |
| **Hematologic** | 55 (1.3) |
| **Eye** | 39 (0.9) |
| **Genetic** | 25 (0.6) |
| **Renal** | 17 (0.4) |
| **Infections** | 17 (0.4) |
| Among 4,194 (75.1%) patients reporting who attended at least one IHM modality that was not massage therapy. Patients could have had more than one diagnosis during the study period. Bold values are Major Expanded Diagnosis Clusters (MEDC). Indented values are prevelant expanded diagnosis clusters within the MEDC. Abbreviations: MEDC, Major Expanded Diagnosis Cluster; IHM, integrative health and medicine | |
