# Supplementary material for: Implementing paper-based patient-reported outcome collection within outpatient integrative health and medicine
Source: PLoS One. 2024 May 29;19(5):e0303985. doi: 10.1371/journal.pone.0303985 (PMC11135778; doi:10.1371/journal.pone.0303985)
Supplement: S2 Table — (DOCX) [file pone.0303985.s002.docx]

| **Supplemental Table 2: Adjusted Odds Ratios for Completing Any Pre-Encounter PRO** | | | | |
| --- | --- | --- | --- | --- |
| **Category** | **Comparison** | **Estimate** | **95% CI Low** | **95% CI High** |
| Age | Age 18-30 v. 51-60 | 0.825 | 0.673 | 1.012 |
| Age | Age 31-40 v. 51-60 | 0.955 | 0.799 | 1.141 |
| Age | Age 41-50 v. 51-60 | 0.953 | 0.802 | 1.132 |
| Age | Age 61-70 v. 51-60 | 1.036 | 0.871 | 1.232 |
| Age | Age 71+ v. 51-60 | 0.875 | 0.715 | 1.071 |
| Sex | Female v. male | 1.178 | 1.028 | 1.349 |
| Ethnicity | Declined/missing v. NH | 0.877 | 0.705 | 1.091 |
| Ethnicity | Hispanic/Latino v. NH | 1.022 | 0.709 | 1.474 |
| Race | American Ind v. White | 0.670 | 0.224 | 2.005 |
| Race | Asian v. White | 0.994 | 0.578 | 1.710 |
| Race | Black/AA v. White | 1.161 | 0.978 | 1.378 |
| Race | Other/Multi v. White | 1.063 | 0.622 | 1.818 |
| Race | Declined/missing v. White | 1.057 | 0.786 | 1.423 |
| Visits | +1 visit beyond 2 visits | 1.016 | 1.009 | 1.022 |
| Location | Clinic 2 v. Clinic 1 | 0.060 | 0.047 | 0.075 |
| Location | Clinic 3 v. Clinic 1 | 0.729 | 0.605 | 0.877 |
| Location | Clinic 4 v. Clinic 1 | 1.209 | 0.990 | 1.477 |
| Time | 2019Q2 v. 2019Q1 | 4.193 | 3.584 | 4.904 |
| Time | 2019Q3 v. 2019Q1 | 3.223 | 2.752 | 3.774 |
| Time | 2019Q4 v. 2019Q1 | 3.668 | 3.115 | 4.318 |
| Time | 2020Q1 v. 2019Q1 | 2.072 | 1.750 | 2.454 |
| Time | 2020Q2 v. 2019Q1 | 0.148 | 0.124 | 0.177 |
| Time | 2020Q3 v. 2019Q1 | 0.163 | 0.133 | 0.199 |
| Chief compaint | Pain complaint (yes v. no) | 1.365 | 1.215 | 1.534 |
| Chief compaint | Headache complaint (yes v. no) | 1.066 | 0.877 | 1.295 |
| Chief compaint | Anxiety complaint (yes v. no) | 3.188 | 2.601 | 3.908 |
