# Supplementary material for: Implementing paper-based patient-reported outcome collection within outpatient integrative health and medicine
Source: PLoS One. 2024 May 29;19(5):e0303985. doi: 10.1371/journal.pone.0303985 (PMC11135778; doi:10.1371/journal.pone.0303985)
Supplement: S3 Table — (DOCX) [file pone.0303985.s003.docx]

| **Supplemental Table 3: Adjusted Odds Ratios for Completing Any Paired PRO** | | | | |
| --- | --- | --- | --- | --- |
| **Category** | **Comparison** | **Estimate** | **95% CI Low** | **95% CI High** |
| Age | Age 18-30 v. 51-60 | 0.861 | 0.677 | 1.095 |
| Age | Age 31-40 v. 51-60 | 1.279 | 1.041 | 1.572 |
| Age | Age 41-50 v. 51-60 | 0.939 | 0.768 | 1.147 |
| Age | Age 61-70 v. 51-60 | 1.077 | 0.879 | 1.319 |
| Age | Age 71+ v. 51-60 | 0.841 | 0.664 | 1.066 |
| Sex | Female v. male | 1.026 | 0.879 | 1.198 |
| Ethnicity | Declined/missing v. NH | 0.845 | 0.652 | 1.096 |
| Ethnicity | Hispanic/Latino v. NH | 1.285 | 0.826 | 1.999 |
| Race | American Ind v. White | 0.359 | 0.091 | 1.413 |
| Race | Asian v. White | 0.853 | 0.421 | 1.729 |
| Race | Black/AA v. White | 1.109 | 0.919 | 1.339 |
| Race | Other/Multi v. White | 0.705 | 0.394 | 1.260 |
| Race | Declined/missing v. White | 1.273 | 0.900 | 1.800 |
| Visits | +1 visit beyond 2 visits | 1.039 | 1.031 | 1.047 |
| Location | Clinic 2 v. Clinic 1 | 0.377 | 0.262 | 0.543 |
| Location | Clinic 3 v. Clinic 1 | 1.322 | 1.088 | 1.607 |
| Location | Clinic 4 v. Clinic 1 | 1.146 | 0.874 | 1.504 |
| Time | 2019Q2 v. 2019Q1 | 0.711 | 0.600 | 0.843 |
| Time | 2019Q3 v. 2019Q1 | 1.082 | 0.906 | 1.293 |
| Time | 2019Q4 v. 2019Q1 | 2.505 | 2.087 | 3.007 |
| Time | 2020Q1 v. 2019Q1 | 0.964 | 0.790 | 1.176 |
| Time | 2020Q2 v. 2019Q1 | 0.165 | 0.122 | 0.225 |
| Time | 2020Q3 v. 2019Q1 | 0.994 | 0.736 | 1.343 |
| PrePRO | Any pre-score ≥ 4 (yes v. no) | 0.953 | 0.856 | 1.061 |
| Chief compaint | Pain complaint (yes v. no) | 0.791 | 0.692 | 0.904 |
| Chief compaint | Headache complaint (yes v. no) | 1.191 | 0.956 | 1.483 |
| Chief compaint | Anxiety complaint (yes v. no) | 1.020 | 0.769 | 1.352 |
